# Low-Cost and Durable Bipolar Plates for Proton Exchange Membrane Electrolyzers

**DOI:** 10.1038/srep44035

**Published:** 2017-03-15

**Authors:** P. Lettenmeier, R. Wang, R. Abouatallah, B. Saruhan, O. Freitag, P. Gazdzicki, T. Morawietz, R. Hiesgen, A. S. Gago, K. A. Friedrich

**Affiliations:** 1 Institute of Engineering Thermodynamics, German Aerospace Center, Pfaffenwaldring 38-40, Stuttgart, 70569, Germany; 2 Hydrogenics Corporation, 220 Admiral Boulevard, Mississauga, ON L5T 2N6, Canada; 3 Institute of Materials Research, German Aerospace Center, Linder Hoehe, 51147, Cologne, Germany; 4 University of Applied Sciences Esslingen, Dept. of Basic Science, Kanalstrasse 33, 73728, Esslingen, Germany; 5 Institute of Energy Storage, University of Stuttgart, Stuttgart, 70550, Germany

## Abstract

Cost reduction and high efficiency are the mayor challenges for sustainable H_2_ production via proton exchange membrane (PEM) electrolysis. Titanium-based components such as bipolar plates (BPP) have the largest contribution to the capital cost. This work proposes the use of stainless steel BPPs coated with Nb and Ti by magnetron sputtering physical vapor deposition (PVD) and vacuum plasma spraying (VPS), respectively. The physical properties of the coatings are thoroughly characterized by scanning electron, atomic force microscopies (SEM, AFM); and X-ray diffraction, photoelectron spectroscopies (XRD, XPS). The Ti coating (50 μm) protects the stainless steel substrate against corrosion, while a 50-fold thinner layer of Nb decreases the contact resistance by almost one order of magnitude. The Nb/Ti-coated stainless steel bipolar BPPs endure the harsh environment of the anode for more than 1000 h of operation under nominal conditions, showing a potential use in PEM electrolyzers for large-scale H_2_ production from renewables.

In 1973, Russell and coworkers introduced solid polymer electrolyte (SPE) (also known as proton exchange membrane; PEM) electrolysis for the production of hydrogen from water splitting^1^. Remarkably, back then they proposed the idea of using the hydrogen as energy storage for off peak electricity grid periods as well as the possibility for its distribution and use for automotive applications. These are nowadays the main drivers for PEM electrolysis technology^2^,^3^,^4^. Neither the use of H_2_ as large-scale energy storage, nor the use of hydrogen in the automotive sector is yet established. The reasons are the still limited share of renewables in the power sectors, the delayed commercialization of fuel cell cars and the corresponding hydrogen refueling infrastructure. In addition, a factor contributing dominantly to high cost of hydrogen produced by sustainable methods such as electrolysis is the high electrical energy cost^2^.

In this context, there are two possibilities for reducing the hydrogen production costs by PEM electrolysis^4^,^5^. The first one is to decrease the operation costs (OPEX) by increasing efficiency and reducing electricity costs. The second possibility is to decrease the capital expenditure (CAPEX) of electrolysis, which is prominent in all Power-to-Gas plants, by reducing the amount of expensive materials, increasing power density or optimizing the balance of plant (BOP). For the first approach, the increase of efficiency is equivalent to reduction of overpotential, which correspond mostly to the sum of the ohmic contributions of electrical nature (interconnectors) and ionic conductive parts (membrane, ionomer), as well as the activation overpotential of the oxygen and hydrogen evolution reactions (OER, HER)^6^. The main focus of research groups is currently on the optimization of the OER, along with the reduction of precious metal content in the electrodes, as both have a major impact on the overall efficiency^7^,^8^,^9^,^10^,^11^,^12^,^13^. For reducing the ohmic resistance, the SPE can be modified or reduced in thickness. The electrical properties and contact between the interconnectors, namely bipolar plates (BPP) or current collectors (CC), and the electrodes can be improved as well by using precious metal coatings^14^,^15^,^16^ or by optimizing other functional properties^17^,^18^,^19^. However, these coatings are quite expensive due to chemical processing, cleaning and etching procedures (e.g. dimensional stable anodes, DSA); and the use of large amount of expansive and scarce metals such as Ir and Pt.

The second approach aims to reduce the specific hydrogen production costs by decreasing CAPEX. Most of the R&D activity is focused on improving stack cost effectiveness, since it is responsible for ca. 60% of the system costs^4^. The bulk of the stack costs is caused by the BPP and CC^2^,^4^, which are made of titanium. Especially the highly corrosive environment at the anode site^20^ necessitates the use of titanium, which is costly and difficult to machine^21^. The use of stainless steel can significantly lower the manufacturing costs^14^,^20^, especially for complicated flow field designs^22^. However, it needs to be protected with a corrosion resistant coating due to the highly oxidative anode environment and local contact with the acidic membrane^14^,^23^,^24^,^25^. Substantial efforts in European projects such as NOVEL^26^, NEXPEL^27^, COATELY^28^, etc. have been dedicated to replace Ti as base material by coated stainless steel.

Several groups have investigated the implementation of either Ti or stainless steel BPPs with surface coatings over the last decades. Often additional precious metal surface modifications are used for reducing the contact resistance and thus increasing the efficiency. Especially, the use of Pt^14^ and Au^29^ as surface modifiers to reduce contact resistance may end up in significant higher material costs. Niobium may be used as an alternative to Pt based on the superior corrosion protecting properties and stable behavior in acid environment^30^,^31^. Niobium coatings for bipolar plates of PEM water electrolyzers were investigated for the first time by Russell and co-workers^32^. Compared to Pt and Au, the abundance of Nb in the earth crust is in the same order of magnitude as other industrial relevant materials such as Cu, Sn or Zn^33^. The present price range of Nb (50–90 €/kg) is significantly lower than that of Pt or Au with ca. 27.000 €/kg and 35.000 €/kg, respectively (Finanzen.net, July 2016)^34^.

We report the development of a non-precious metal based, corrosion resistant and highly conductive coating of Nb/Ti for stainless steel bipolar plates. It allows reducing the production costs of bipolar plates, lowering the amount of titanium in general, and decreasing the interface contact resistance (ICR). A systematic physical and electrochemical characterization of the coating was carried out and the study was finalized with a 1000 h durability test in a high-end commercial PEM electrolyzer stack without showing any degradation.

## Discussion of Results

### Physical and electrical properties

**Figure 1:**
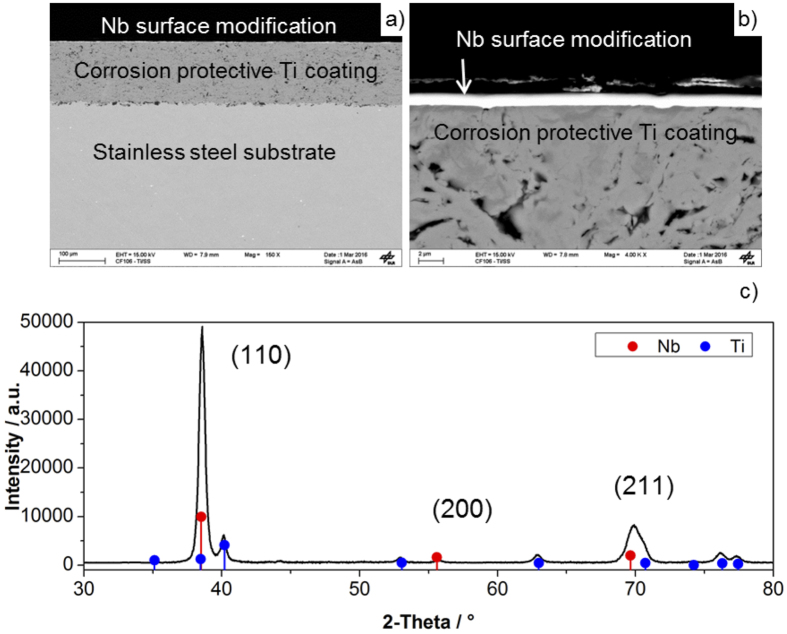
**(a)** Low and (**b**) high magnification cross-section SEM images, and (**c**) XRD spectrum of Nb/Ti/ss.

The morphology of the Nb/Ti coatings was investigated by SEM technique. Figure 1a shows an overview of the Nb/Ti/ss sample, highlighting the differences between the bulk substrate and thermally sprayed Ti coating, which is approximately 130 μm thick. Figure 1b presents a close-up of the interface between the much thinner Nb layer and Ti. The dense splat structure of the Ti coating is clearly evident from the SEM image. The top layer of Nb has an average thickness of 1 μm and covers completely the surface of the Ti coating. Figure 1c shows the XRD spectrum of Nb/Ti/ss. Most of the X-ray radiation is absorbed and diffracted by the Nb coating, while the peaks corresponding to the Bragg planes of Ti are barely visible. The X-rays did not penetrate the stainless steel substrate. The Nb coating is preferably oriented as (110) crystals and lattice parameters from XRD were calculated by Rietveld refinement analysis. A summary of the results is presented in Table 1. The crystallite size has been calculated to be between 10 and 20 nm which is well supported by AFM analysis (Figure S4.3d) and is in good agreement with literature data for lattice parameter *a* of around 3.3 Å^35^. Neither TiO_2_ nor Nb_2_O_5_ phases were observed, accounting for the high phase purity of the Ti and Nb coatings, respectively.

**Figure 2:**
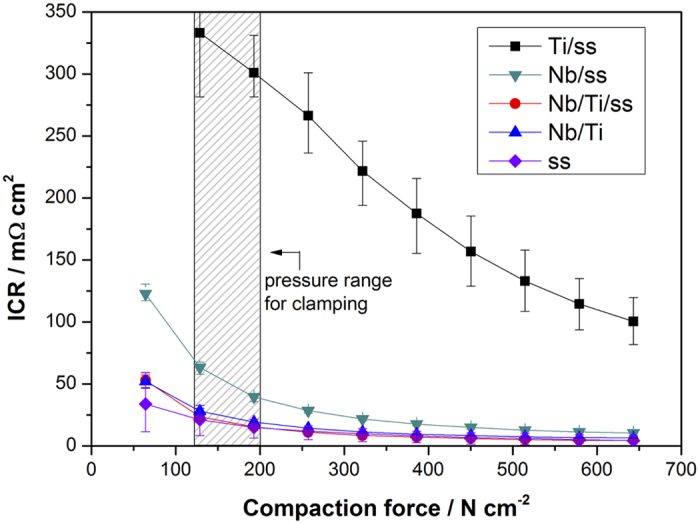
Interfacial contact resistance (ICR) *vs.* Compaction force of Ti/ss, Nb/ss, Nb/Ti/ss, Nb/Ti and ss. The shaded area of the X-axis corresponds to the compression forces used for clamping the test cells in the PEM electrolyzer stack for the 1000 h test.

The natural passivation of the Ti leads to the formation of amorphous semi-conducting TiO_2-x_ on its surface^36^ and thereby an additional coating of Au^29^ or Pt^16^,^37^ is necessary to maintain a low contact resistance. In this work Nb has been chosen for this purpose given its large abundance and lower cost compared to the precious metals. To show the improved electrical and interfacial contact behavior of this surface modification, ICR measurements were performed under different compaction forces, Fig. 2. Almost one order of magnitude lower ICR is observed for Nb/Ti/ss compared to Ti/ss. In addition, Nb/Ti/ss and Nb/Ti sample show lower ICR than Nb/ss as a result of improved adherence of Nb on Ti than on stainless steel. In fact, Nb surface modification has a positive impact on the electrical properties of titanium oxide^36^. The ICR of Nb-modified Ti coating is lower than 25 mΩ cm^2^ at compacting pressures of more than 120 N cm^−2^, which is the common pressure range for assembling a PEM electrolyzer stack. The compression forces used for clamping the test cells in the PEM electrolyzer stack for the 1000 h test are indicated as a shaded interval of the X-axis in Fig. 2. The ICR of bulk stainless steel is included in Fig. 2. One can realize from our results that the protective layers do not reduce significantly the electronic conductivity of bulk stainless steel. It is worthwhile noting that the ICR results of Fig. 2 are not influenced by the roughness of the Nb/Ti coatings. The arithmetic average (R_a_) and root mean squared (R_q_) roughness were determined. For all samples R_a_ and R_q_ are 7.7 ± 0.8 nm and 6.1 ± 0.6 nm, respectively, which corresponds to surface state of the magnetron sputtered PVD coating of Nb, as the substrate, either ss or Ti coating, was sanded finely prior deposition.

Lastly, there is no significant difference in ICR between Nb/ss and Nb/Ti/ss. However, as it will be shown in the following section, a thin film of Nb on stainless steel is inappropriate because its properties are not sufficient for protecting stainless steel against pitting corrosion in the acid environment. A thick and robust coating of Ti is required for this purpose. Each coating has different function and both of them are necessary: (i) Nb reduces ICR of the Ti coating and (ii) the Ti coating prevents corrosion of stainless steel.

### Electrochemical half-cell measurements

**Figure 3:**
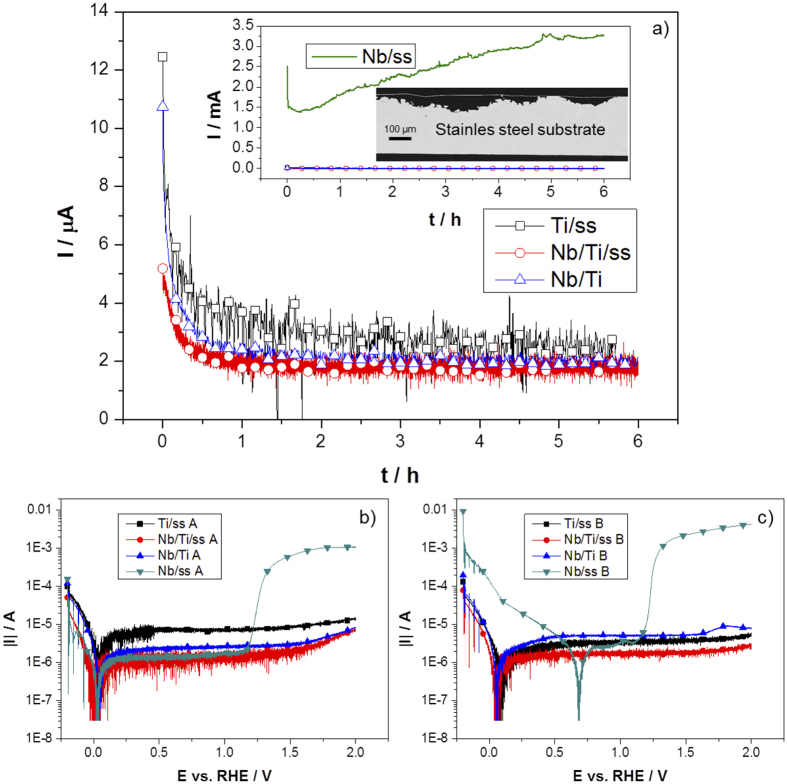
(**a**) Chronoamperometric measurements at 2 V *vs.* RHE constant potential of Nb/Ti/ss and Nb/Ti; For comparison, the inset shows current transient characteristic of Nb/ss (green) and a cross-section SEM image of the sample after the measurement. Potentiodynamic characteristics from 0.2 to 2 V *vs.* RHE of all samples before (A) and after (B) the chronoamperometric measurements are presented in (**b**) and (**c**), respectively. Measurements were carried out in O_2_-saturated condition, 0.5 M H_2_SO_4_, 65 °C.

This section presents the electrochemical behavior and stability of the samples under accelerated stress test (AST) conditions, namely pH = 0, at 65 °C and a *E*_*const*_ of 2 V *vs.* RHE^25^. Figure 3a shows the chronoamperometric measurements applying this potential for 6 h of Ti/ss, Nb/Ti and Nb/Ti/ss. For comparison, the inset presents the results of the three samples and Nb/ss with a larger scale of the y-axis showing the staggering differences in their current transients. The two thermally sprayed Ti-coated samples, with and without Nb coating, and Nb/Ti show similar behaviour during the 6 hours of constant polarization. The samples of Nb/Ti/ss and Nb/Ti exhibit slightly lower oxidation current than Ti/ss, proving that the Ti coating is sufficient for protecting the stainless steel substrate, while the Nb layer alone is not enough.

More electrochemical information can be obtained by sweeping the applied potential from −0.2 to 2 V *vs.* RHE. Figure 3b and c depict the potentiodynamic measurements before and after the chronoamperometric measurements of Fig. 3a, respectively. The electrochemical corrosion parameters such as *E*_*corr*_ and *i*_*corr*_ were calculated from the electrochemical characteristics and the obtained values are summarized in Table 2. Except for Nb/Ti, the *i*_*corr*_ of all Nb-coated samples lie below 1∙10^−6^ A cm^−2^ before and after the chronoamperometric test. Conversely, Ti/ss shows an order of magnitude higher *i*_*corr*_ as a result of the formation of TiO_2−x_. The results clearly indicate that the surface reaction of the particular material is predominantly the oxidation reaction of Nb and Ti. *E*_*corr*_ is located around 0 V for every initial potentiodynamic curve and the cathodic reaction corresponds to hydrogen evolution. Subsequently, a wide passivation potential window develops up to 1.1 V *vs.* RHE. However, at higher potentials the Nb/ss sample exhibits the typical trans-passivation wave of stainless steel, which corresponds to oxidation of Fe^2+^ to Fe^3+ ^38,39. The effect is clearly evident in the current transient of Nb/ss presented in the inset of Fig. 3a, which shows a steady increase of the oxidation/dissolution current density. At this potential, the Cr-Fe beneath the Nb coating dissolves as consequence of pitting corrosion increasing largely the electrochemical surface area of the electrode, SEM image of inset of Fig. 3a.

Furthermore, Fig. 3c shows how the *E*_*corr*_ shifts for the Nb/ss sample for more than 400 mV while for the other specimens it remains practically unchanged. As expected, *i*_*corr*_ is not changing in value for this sample, but the current density at the trans-passive region increased largely, which is associated with the increase of the electrochemical surface area after the chronoamperometry test. Additionally, a significant increase of the cathodic current can be observed. The two different slopes in this region indicate a reduction reaction process, which takes place between 0 and 0.7 V *vs.* RHE, just after the H_2_ evolution. In conclusion, the thin Nb coating is not sufficient for protecting the stainless steel and cannot be used as a corrosion protective layer. In contrast, the Ti-coated samples show no pinholes at all or any visible degradation indications after the chronoamperometric measurements (Figure S3).

### Physical characterization after AST compared to initial

**Figure 4:**
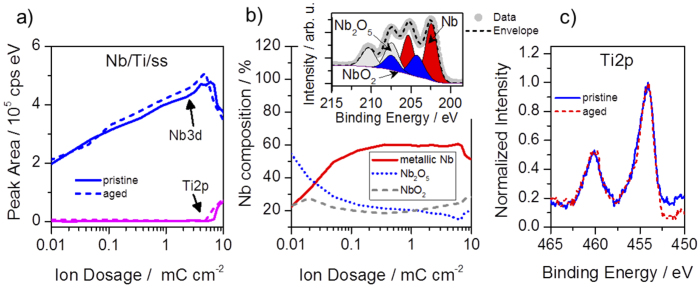
(**a**,**b**) and (**c**) show XPS depth profile analysis of Nb/Ti/ss. (**a**) peak area of Nb3d and Ti2p as a function of Ar^+^ ion dosage; (**b**) Nb composition versus Ar^+^ ion dosage. The inset shows the identified Nb species for an ion dose of 0.05 mC cm^−2^; (**c**) Ti2p spectra of the pristine and the degraded sample after applying an ion dosage of ~7–8 mC cm^−2^.

In order to determine possible material changes before and after the electrochemical tests, XPS analysis was carried out. Since the XPS results of the samples are similar in Fig. 4a,b and c, only results of Nb/Ti/ss are shown as an example. The depth profiles showing the Ti2p and Nb3d peak areas versus ion dosage are provided in panel (A). The area of the Ti2p peak is zero until the Nb layer is removed around an ion dosage of ~7 mC cm^−2^. Concurrently, the intensity of the Nb3d peaks starts to decrease rapidly around 7 mC cm^−2^. This behavior is common for both, the pristine and the aged Nb/Ti/ss samples and is also observed analogously for Nb/ss and Nb/Ti. The Nb3d XPS spectra can be deconvoluted to indicate three species of niobium, namely metallic Nb, NbO_2_ and Nb_2_O_5._ The deconvolution is performed by fitting three doublets of peaks with the low energy peaks (Nb3d_5/2_) located at binding energies of 202.6, 204.4 and 207.5 eV, respectively^40^,^41^,^42^, as demonstrated in the inset of Fig. 4b. The Nb composition profile in panel b reveals that the surface layer of the Nb coating is dominated by Nb_2_O_5_; at the very surface, i.e. before the first sputter steps, the concentration of metallic Nb is zero (Figure S2). While a decrease of Nb_2_O_5_ is observed with depth, the concentration of NbO_2_ is roughly constant at 20–30%. After the Nb_2_O_5_ amount reaches a constant value upon etching, the fraction of metallic Nb equals to ~ 60%. It was suspected that as a result of aging, Ti oxide may build up on the Nb/Ti interface. However, no evidence of the presence of Ti oxide was found in any of the analyzed samples. Rather Ti2p_3/2_ peaks positions of pristine and aged samples at 545 and 460 eV clearly correspond to metallic Ti^40^.

Niobium oxides have received a significant attention due to their applications e.g. for catalysis, biomaterials, sensors, optics, and microelectronics. The stable oxides, namely, niobium monoxide (NbO, conductor), niobium dioxide (NbO_2_, semiconductor), and niobium pentoxide (Nb_2_O_5_, insulator) have very different properties, however, the Nb-O system is complicated by the presence of numerous metastable oxides NbO_x_ with 0 < x < 1 and 2.0 < x < 2.5 as well as by the existence of a multitude of Nb_2_O_5_ polymorphic modifications^43^. In particular, anodic Nb_2_O_5_ formed in acidic electrolytes is reported to be highly disordered with oxygen deficiencies and exhibiting n-type semiconducting properties^44^.

In order to obtain some additional information about the conductivity of the materials before and after the electrochemical tests, AFM measurements with lock-in current amplifier (Peakforce TUNA) and Pt/Ir-coated AFM probes were performed. On the Nb/Ti/ss samples, no evidence of corrosion or damage of the Nb coating was detected. No change in conductive area before (Figure S4.2b) and after AST was observed (Figure S4.2d) at an applied voltage of U = 3 V. The surface of the Ti pre-coated samples stayed without change in contrast to the samples with an Nb layer coated on ss (Figure S4.3). The relative thickness of the Nb_2_O_5_ is however increasing after the AST as shown in (Figure S2a and b) as well as the metal vs. oxide ratio (Figure S2c) for the Nb/Ti/ss sample, which results in decreased electrical conductive properties since stoichiometric Nb_2_O_5_ is not conductive^45^. This result is in good agreement with the increasing bandgap from 1.3 to 2.2 eV measured by i(V) curves with AFM.

### 1000 h test in a commercial PEM electrolyzer

**Figure 5:**
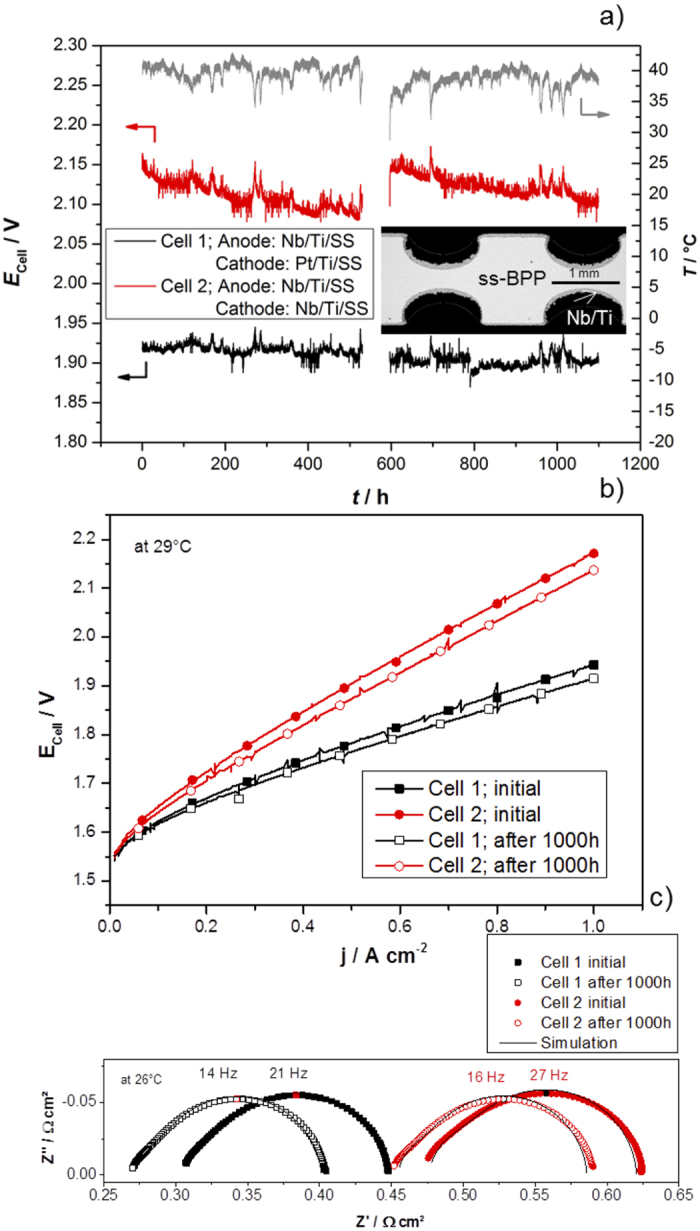
(**a**) Long-term test at 1 A cm^−2^ of the Nb/Ti coatings on stainless steel bipolar plates, at ca. 38 °C and 6.5 × 10^5^ Pa balanced pressure, carried out in 0.75–2.5 Nm^3^ H_2_ h^−1^ Hylyzer Hydrogen Generator, Hydrogenics; Inset: a SEM picture of the Nb/Ti/ss bipolar plate after the mentioned long-term test; (**b**) Current potential curve of cell 1 and cell 2 before and after the 1000 h test, from 0.01 A cm^−2^ to 1 A cm^−2^ at ca. 29 °C and a scanning rate of 4 mA cm^−2^ s^−1^; (**c**) Nyquist plot and the simulation of cell 1 and 2 before and after the 1000 h long term test at 26 °C, 20 A, an amplitude of 3 A and a frequency range of 1 kHz to 100 mHz.

It has been shown in the previous sections that the Nb/Ti coating combines excellent corrosion protection to stainless steel and improved electric surface properties can be obtained without any precious metal layers. Since the Nb/Ti coating meets the technical and commercial requirements, we carried out a performance as well as the durability evaluation at 1 A cm^−2^ in a commercial 120 cm^2^ active area PEM electrolyzer short stack for more than 1000 h at an average temperature of 38 °C. The temperature profile of the stack corresponds to the right Y-axis of Fig. 5a. The temperature results only by efficiency loss in the stack, which heats up the entire system. At 1 A cm^−2^ no higher temperatures could be reached. The temperature of the commercial hydrogen generator used for the longevity tests depends strongly on the applied current density.

It needs to be mentioned, that these conditions are rather conservative and therefore they cannot be considered as accelerating stress tests (AST). If the tests would have been carried out at 2 A cm^−2^, then a stack temperature of approx. 55–60 °C would be achieved^46^. Yet, we opted for a more conservative 1 A cm^−2^ due to the following. Alkaline electrolyzers operate at current densities of 0.2–0.4 A cm^−2 ^47,48. The reason of operating PEM electrolyzers at high current densities is driven mainly by stack cost reduction and low footprint, although efficiency and durability are compromised^46^,^49^. There would not be need for operating PEM electrolyzers at high current densities the moment its investment cost can rival the alkaline technology. Future PEM electrolyzers are expected to have low cost stack materials such as stainless steel interconnectors, non-precious metal coatings, hydrocarbon membranes, and low PMG loading MEAs. Thus large facilities might operate at low current densities as well as under other less stressing conditions such as temperature. Operating PEM electrolyzers at low or high current densities is still debatable.

Figure 5a shows the long-term performance of two cells with stainless steel bipolar plates. Both cells were measured with the same configuration in the same short stack, but cell 1 was coated with Nb/Ti on the anode side and Pt/Ti on the cathode side^14^,^22^, while cell 2 had Nb/Ti coatings on both sides. The choice of varying the coating on the cathode side can be justified as follow. In our previous reports we demonstrated that the Pt/Ti coatings on stainless steel BPPs are suitable for both anode and cathode, achieving a performance comparable to the reference cell^22^. Furthermore, we clearly showed the need of a highly conductive coating resistant to H_2_-embrittlement on the cathode side. For this reason we decided to apply the Nb/Ti coating on the anode and cathode sides of cell 2.

The inset of Fig. 5a presents a cross-section SEM image of the BPP. Prior deposition of the Nb coating, the Ti coating was sanded in different steps until achieving a smooth surface. This procedure allows achieving a uniform thickness (Figure S6a) of the Ti coating deposited on the contact area of the flow field of the BPP, including etches and corners of the channels. Conversely, the thickness of the Ti coating is lower in the rib walls than in the area parallel to the contact surface. However, applying a conductive coating of Nb/Ti inside the channels is not necessary and should be avoided by using a mask. The surface of the Nb coating follows the corresponding area of the Ti coating and if the Nb target is sufficiently large, the thickness of the thin film coating should be quite uniform on the contact area of the BPP.

While cell 1 showed a stable performance, cell 2 rapidly increased its potential after ca. 500 h of operation causing large differences in performance. Figure 5b depicts the current-potential characteristics of the two cells before and after the long-term measurements. First, one can observe that the Nb coating in the cathode side has a detrimental effect in the cell performance since the anode side coating is the same for both cells. The 92E stack is quite reliable for achieving similar performance of cells having the same MEA and other cell components^46^,^49^. Therefore any other reason for the differences in performance between cell 1 and 2 but the coatings applied on the BPPs can be discarded. Additional information can be gained by analyzing the EIS shown in Fig. 5c. Unfortunately the different electrochemical tests presented Fig. 5a,b and c cannot be performed at the same temperature. As mentioned above, these were carried out on a commercial hydrogen generator, which does not permit controlling the operation temperature. Neither the stack nor the water reservoir has a heating/cooling system. The stack reaches certain temperature depending on the operation current density. To help the reader to compare our results with others reported at different temperatures, an accurate simulation of the polarization curves and EIS produced by a well-validated mathematical scripted model has been included in the Supporting Information. The model is able to simulate the polarization curves by taking the measured stack temperature and pressure profiles as input parameters. The electrochemical parameters as well as overpotentials and impedances are provided by the EIS measurements. Details about this model are presented in the Supporting Information.

The ohmic resistances measured by EIS are compared with the slopes of the polarization curves by using the validated model presented in the Supporting Information. The low frequency impedances on the real axis are in good agreement with these slopes. However, the analysis of the slope of the polarization curves of the test station is highly sensitive to the chosen current density range. Small changes in pressure and temperature during the measurement affect the slope. Therefore, the temperature and pressure profiles were used as an input parameter for the model. All other parameters were taken from the EIS analysis. The simulated polarization curves fit well with those measured, Figure S5c, which indicates a good consistency of the different measurement methods.

The impedance spectra in Fig. 5c indicate a pronounced increase of the ohmic resistance for cell 2. The increase in resistance can be due to hydrogen embrittlement of Nb combined with oxidation reactions, which seems to become a problem with hydrogen access^50^,^51^, since the formed hydrides are not stable^52^. Second, the increase in resistance caused by formation of Nb and Ti amorphous oxides on the cathode side cannot be excluded. The formations of oxides on the Ti of the cathode side, beneath the delaminated Nb coating, can occur during stand-by periods or when the PEM electrolyzer is turned off^22^,^37^. The effect can be clearly noticed when the electrolyzer was turned of after 500 h, Fig. 5a. Possible reasons of this degradations are: (i) natural passivation of Ti in the areas where Nb has been delaminated; (ii) formation of a galvanic pair between Pt (noble metal) from the cathode catalyst and Ti (non-noble metal); (iii) oxidative environment due increase of O_2_ concentration on the cathode due to crossover; (iv) sudden operation as H_2_/O_2_ (from crossover) PEM fuel cell due to oxygen reduction reaction (ORR) on Pt; and (v) parasitic currents and capacitor discharges originated from the electronics which polarize negatively the stack. It is worthwhile noting that even a thin passive layer of TiO_x_ on cathode side of the BPP has much more detrimental effect than the anode side due to the different nature of current collectors of each electrode.

Post mortem SEM analyses of the bipolar plates show for the anode side a well-attached Nb thin film on top of the Ti coating. The Pt coating on the cathode side has shown good attachment on Ti^22^. However the Nb coating on the cathode side presents cracks and delamination (Figure S6c), which certainly will lead to oxidation of Ti below the Nb, deteriorating the electrical properties and consequently decreasing cell performances. In fact, both cells have similar performance at the very beginning of the activation protocol (Figure S8). The rapid increase of voltage of cell 2 indicates clearly an early degradation mechanism, which can explain the differences in performance between cell 1 and cell 2 at the beginning of the long-term test. The Nb delamination on the cathode side of cell 2 was observed with the naked eye after disassembling the stack. Moreover, Fig. 5a and b show a progressive increase in performance (decrease of *E*_*cell*_) of these cells compared to the initial one. This phenomenon was previously observed for other stacks as well and the increase is mostly attributed to MEA aging effects^46^, which suddenly stop after the first 500 h (Figure S5a) in which the PEM electrolyzer unit was shut-down on purpose.

Recapitulating the results show the positive impact of Nb as an effective surface modification of titanium coatings for the anode side regarding efficiency and durability in a 1000 h test. XPS analysis of the DI water resin of the anode site recirculation cycle, where all ions of the anode side accumulate showed no significant increase of Fe. Taking into account the molecular weight of Fe and Ti, Fe ions at 0.1 wt.% were threefold lower than Ti ions (ca. 0.3 wt.%). Since both values are extremely low they demonstrate the protection by the Ti coating. Furthermore, the iron detected may also be leached out from tubes, valves, etc. It is obvious, that the use of Nb at the cathode side is not beneficial, but thanks to the well conductive behavior of stainless steel in hydrogen environment^53^,^54^, the need of any coating for the cathode side can be discussed in general since stainless steel is state of the art to be in service with hydrogen^55^. However, several solutions, like graphitic or nitride coatings, exist for PEMFC and may be suitable also for electrolysis^56^.

### Coating cost aspects

A detailed cost estimation of the thermally sprayed Ti coating for large production of bipolar plates for megawatt electrolyzer systems with large surface area is provided elsewhere^14^. The cost was estimated to be around 3.13 USD per BPP considering a production facility exclusively dedicated for this purpose. Such coating facility would be realistic taking into account that the chemical and refinery industries in Germany will require hundreds of TWh a^−1^ electrolyzers, if all the H_2_ they currently use would come from renewables, and not from hydrocarbons. However, it is difficult to determine accurately a price per BPP since the coating cost in the industry will be largely different compared to a research center. In addition, coating of large number of BPPs in a day or in one run reduces the cost further.

Regarding the cost of the Nb coating, essentially, target material costs and coating rates are the primary factors, which define the coating cost. To compare the cost of Nb with that of Pt and Au under the same deposition technique, it is acceptable to compare the target material costs and the deposition rates. Pt and Nb coatings have similar deposition rates. Thus, under the application of the same deposition technique (e.g. PVD), Nb-coatings deposited from the relatively lower priced Nb targets will be cheaper. For comparison, a Pt target is 27-fold more expensive than a target of Nb of similar size and purity^57^. Regarding the coating area, industry-sized equipment is fairly in the position of coating surfaces such as 20,000 cm^2^. For example, the coating technique is widely used for coating large area crystal windows. Nevertheless, the number of the coated BPP per day will possibly be in this case reduced and thus the coating cost will be increased. For instance, if it is possible to carry out 5 runs in a day (each run contains a cycle of loading, evacuating, coating, unloading) and at each run, up to 10 small sized BPPs can be loaded and coated, as a result, 50 small BPP will be coated, whereas only 5 large BPPs will be manufactured in a day.

Thick coatings of Nb are also interesting to consider. However, magnetron-sputtering PVD is normally used for producing thin films. Producing a thick coating of Nb by this technique would require long deposition times and consume large amount of electricity. VPS is more suitable for producing thick and robust coatings of Nb. The development of a dense Nb coating by VPS is part of our ongoing work and the results will be reported in a separate work.

## Conclusions

In this work, we have developed a non-precious dual-layer coating for protecting stainless steel based bipolar plates (BPP) in the highly corrosive environment of PEM electrolyzers. These BPPs are expected to have much lower cost than the state-of-the-art Ti-based ones coated with Pt or Au, which are the most expensive component of the stack^14^. Successive steps of VPS (Ti, 50 μm) and PVD magnetron sputtering (Nb, 1 μm) were employed to deposit Nb/Ti layers on stainless steel substrates. Electrochemical accelerated stress tests in acid environment verified the stability of the coatings, protecting the stainless steel from corrosion. The Nb surface modification decreased the contact resistance by one order of magnitude compared to uncoated Ti. The electrical properties of Nb/Ti did not deteriorate in spite of the formation of the mixed and oxygen deficient Nb_2_O_5−x_ on Nb (AFM and XPS). A 1000 h test at 1 A cm^−2^ in a commercial PEM electrolyzer showed stable cell performance when coating the stainless steel BPPs on the anode side. However, the Nb layer experienced cracking and delamination due to H_2_ embrittlement, suggesting only the anode side needs to be coated. No corrosion of the stainless steel was observed at end of the long-term test. The use of non-precious metal-coated stainless steel bipolar plates in PEM electrolyzers is possible, rendering the technology more profitable for sustainable large production of H_2_.

Needless to mention that a 1000 h durability test can serve for demonstrating a novel, though high risk, proof of concept. Yet, the test does not demonstrate that the coatings can be used commercially. Rather it shows a promising solution to the high cost of titanium-based BPP with precious metal coatings. It is by any means a decisive test but we expect that the outcome will encourage the industry and academy to perform a more exhaustive evaluation of the coatings.

## Methods

### Coating deposition

The corrosion protection coating of Ti was deposited by vacuum plasma spraying (VPS) as reported elsewhere^14^,^22^. Subsequently, the surface of Ti was modified with Nb (Nb/Ti) by magnetron sputtering physical vapour deposition (PVD). Prior deposition of the Nb coating, the Ti coating is sanded with different grades of SiC, without using any abrasive agent, until achieving a smooth surface with SC4000 sand paper. The same process was applied for the reference stainless steel substrates. The metallic Nb coatings were deposited by magnetron sputtering process using an industry sized gas-flow assisted coater (MEGA, SYSTEC of Co. SVS, Germany), which can accommodate 4 targets of 100 × 200 mm enabling deposition of complex compounds. Prior to coating with Nb, the substrates were etched for 20 minutes by means of inverse sputter etching with argon plasma using 500 V pulse bias voltage. Then the Nb layers were deposited by applying a power of 1.0 kW for 15 minutes to obtain a layer thickness of 1.4 μm. The coatings were deposited 20 cm^2^ active BPP (stack model 92E, Hydrogenics). The BPP have a complex flow field design, which is proprietary information of Hydrogenics and cannot be disclosed.

### SEM, AFM, XRD, XPS analyses

Cross-section backscattered electron (BSE) micrographs were recorded with a Zeiss ULTRA electron microscope with an acceleration voltage of 15 kV. The images were recorded before and after the corrosion tests described below. AFM measurements were performed with a Bruker Multimode 8 AFM (Karlsruhe, Germany). The AFM was equipped with an x-y closed loop scanner with open loop z-axis (nPoint, USA) for Peakforce Tapping. The samples were fixed on 12 mm steel discs with silver adhesive glue. For current recording and current-voltage curve measurements, Peakforce TUNA amplifier and PtIr-coated AFM probes were used (PPP-NCHPT, 42 N/m; Nanosensors). For all current measurements a voltage of 3 V was applied between the scanner/sample and the AFM tip. The relative conductive sample area was evaluated using bearing analysis with a threshold of 20 pA. The i(v) curves were ramped from −2 V up to 2 V. For bandgap determination, a threshold of 1 nA was used. The measurements were performed at room temperature and a relative humidity of 20%. The roughness was measured in a Multimode 8 AFM in Peakforce-Tapping mode by evaluating 10 × 10 μm^2^ images for Ti/ss, Nb/ss and Nb/Ti/ss. To determine the surface roughness parameters: arithmetic average (R_a_) and root mean squared (R_q_) of the layers, 21 subareas with a size of 1 × 1 μm^2^ of each measured image were selected. Based on the depth profiles recorded by use of AFM, the roughness of the Nb coated samples was calculated in order to achieve the arithmetic average of the absolute values R_a_.

The X-ray diffraction patterns were measured with the X-ray diffractometer D8 Discover from Bruker combined with an area detector VÅNTEC-2000 in reflection mode under Bragg-Brentano-conditions. The X-rays are emitted by a copper anode with an accelerating voltage of 45 kV and a tube current of 0,650 mA. The diameter of the collimator was 1 mm. It was done 4 frames with an integration time of 180 s for each frame. The starting position was 12° (2-Theta) till a collimator-angle of 46.5° (in 4 steps) was reached.

XPS experiments have been conducted at room temperature in a Thermo Scientific ESCALAB 250 ultra-high vacuum facility with a base pressure of 1 × 10^−9 ^mbar. The Ar^+^-sputtering depth profiles were performed with a Thermo EX05 ion gun. The Ar partial pressure was set to 2–3 × 10^−8 ^mbar yielding a Ar^+^ current of 3–7 μA at an area of 3 × 4 mm^2^ at 2 kV acceleration voltage and 10 mA emission current. XPS measurements an Al Kα X-ray source (Thermo XR4) and a lens mode covering 0.8 mm^2^ surface area were chosen. The sputtering yields are not calibrated. Therefore, the sputter depths are provided as Ar^+^ ion dosage. In each experiment detailed spectra of the C1s, O1s, Ti2p and Nb3d were recorded. Additionally, before sputtering and after the final sputter step a survey spectrum was recorded in the binding energy range 0–1000 eV.

### ICR measurements

Contact resistance measurements were performed in order to gain deep information about the electrical resistance behavior with respect to the compaction pressure. Dummy samples of Ti/ss, Nb/Ti, Nb/ss and Nb/Ti/ss (Coated 1.8 to 1.8 cm^2^) were placed between two Cu cylinders, which were previously polished until mirror finishing. Untreated Toray paper (280 μm thick) without microporous layer (MPL) was used as compressible contact element for the ICR measurements. The Toray paper was placed between the coating and the Cu surface. The sandwich-like arrangement was compressed with a hydraulic press. Measurement were performed by applying a direct current of 5 A by means of a potentiostat (Zahner elektrik IM6) and booster (Module PP240). The applied force was varied from approx. 50 up to 650 N cm^−^^2^. The ICRs of the coatings were calculated according to an electrical circuit of resistors connected in series, which correspond to the contact interfaces. The ohmic drop caused by the Cu/carbon interface was determined by carrying out using only the carbon paper without any coated specimen. The ICR measurements were performed on the pristine and *post mortem* bipolar plates as well. Further details about the ICR measurements are given in the Supporting Information.

### Corrosion evaluation

Corrosion measurements were carried out in a half-cell by using an electrode sample holder in which 1.8 × 1.8 cm^2^ sized squared specimens were mounted exposing 1 cm^2^ active area to 0.5 M H_2_SO_4_ electrolyte solution. A three-electrode assembly consisted of the aforementioned working electrode, a Pt disc and a reversible hydrogen electrode (RHE, HYDROFLEX) were used as counter and reference electrode, respectively. Potentiodynamic and chronoamperometric characteristics were recorded using a potentiostat/galvanostat (Autolab PGSTAT12) in O_2_-saturated environment at 65 °C to simulate the anode side conditions of PEM electrolysis. A protocol of measurements was established for all samples, namely Nb/Ti, Nb/Ti/ss, Ti/ss and Nb/ss. It starts with forward and backwards linear scan voltammetry (LSV) from −0.2 to 2 V *vs.* RHE (scanning rate of 5 mV s^−1^) to clean the surface of the coatings. Likewise, another sweep at 1 mV s^−1^ was carried out to determine corrosion current (*i*_*corr*_) and potential (*E*_*corr*_). A chronoamperometric measurement (6 hours) at 2 V *vs.* RHE followed and lastly a potentiodynamic measurement at 1 mV s^−1^ was performed to the determine changes in the electrochemical properties of the samples.

### PEM electrolyser tests

The Nb/Ti-coated stainless steel bipolar plates were assembled in a 120 cm^2^ active area rainbow 2-cell short stack (model 92E, Hydrogenics). The coatings were evaluated in the anode side of two cells. Both cells had the same well corrosion protected metal-based current collectors on the anodes side, carbon based gas diffusion layers on the cathodes side. Commercial MEAs (E400 Greenerity, Ir-based anode and Pt-based cathode electrodes coated on Nafion N115CS) were used in both cells. The nature of the catalysts is proprietary information of Greenerity and cannot be disclosed. The difference between both cells laid in the cathode side: Cell 1: Pt/Ti-coated stainless steel; Cell 2: Nb/Ti-coated stainless steel, while the BPPs of both cells are coated with Nb/Ti on the anode side.

The stack was tested in a commercial PEM electrolyzer (0.75–2.5 Nm^3^ H_2_ h^−1^ Hylyzer Hydrogen Generator, Hydrogenics). This unit does not allow controlling the operating temperature. Neither the stack nor the water reservoir has a heating/cooling system. The stack reaches certain temperature depending on the operation current density.

Initially, the stack was evaluated at constant 1 A cm^−2^, for a few days, at approx. 38 °C, and 6.5 × 10^5^ Pa balanced pressure system. After this activation time for reaching stable condition, the stack was characterized by recording polarization curves (step rate of 4 mA cm^−2^ s^−1^) up to 1 A cm^−2^ at approx. 28 °C. Likewise, impedance spectroscopy (EIS) at 27 °C, 20 A constant current, an amplitude of 3 A and at frequencies from 0.1 and 750 Hz was carried out. The amplitude is chosen as low as possible but high enough to observe a proper response. In this specific case, 3 A was chosen as the amplitude given the low resistances for the large active surface of 120 cm^2^ cells. After 1000 h constant condition of 1 A cm^−2^, 6.5 × 10^5^ Pa balanced pressure and ca. 38 °C the measurements were repeated at the same operating conditions. An external potentiostat/galvanostat (Zahner elektrik IM6) and booster (Module PP240) was used to perform the EIS. The water quality was controlled constantly and did not reach a resistance lower than 10 MΩ (0.1 μS). The ion-exchange resin (Aldex Chemical Co. LTD), which was placed right before the stack inlet at the anode side circuit, was analyzed before and after the long-term test to determine corrosion products from the bipolar plates.

## Additional Information

**How to cite this article:** Lettenmeier, P. *et al*. Low-Cost and Durable Bipolar Plates for Proton Exchange Membrane Electrolyzers. *Sci. Rep.* **7**, 44035; doi: 10.1038/srep44035 (2017).

**Publisher's note:** Springer Nature remains neutral with regard to jurisdictional claims in published maps and institutional affiliations.

## Supplementary Material

Supplementary Information

## Figures and Tables

**Table 1:** Structural parameters of Niobium and Titanium in the Nb/Ti/ss coating, calculated using a Rietveld analysis.

Property	Nb (in Nb/Ti/ss)	Ti (in Nb/Ti/ss)
Phase	Niobium	Titanium
Space group	Im-3 m	P63/mmc
Lattice parameters		
a (Å)	3.2954 (6)	3.085 (11)
c (Å)	—	4.81 (6)
Cell volume (Å^3^)	35.788 (19)	39.6 (6)
Crystallite size (nm)	18.4 (11)	18 (5)
Crystal density (g cm^−3^)	8.622 (5)	12.04 (17)

**Table 2:** Corrosion current and corrosion potential of Nb/Ti/ss, Nb/Ti and Nb/ss before and after 6 h chronoamperometric measurement at constant 2 V *vs.* RHE.

Sample	Before		After	
	E_corr_	i_corr_/10^−6^_ _A cm^−2^	E _corr_	i_corr_/10^−6^_ _A cm^−2^
Nb/Ti/ss	−0.01717	0.4	0.05	0.25
Nb/Ti	0.070167	1.1	0.02	0.4
Nb/ss	−0.01814	0.3	0.7	0.6
