# Supplementary material for: Low-Cost and Durable Bipolar Plates for Proton Exchange Membrane Electrolyzers
Source: Sci Rep. 2017 Mar 15;7:44035. doi: 10.1038/srep44035 (PMC5353588; doi:10.1038/srep44035)
Supplement: Supplementary Information [file srep44035-s1.pdf]

Supporting information

Low-Cost and Durable Bipolar Plates for Proton Exchange Membrane Electrolyzers

P. Lettenmeier[1], R. Wang[2], R. Abouatallah[2], B. Saruhan[3], O. Freitag[3], P. Gazdzicki[1], T. Morawietz[4], R. Hiesgen[4], A. S. Gago[1,*], K. A. Friedrich[1,5]

[1]*Institute of Engineering Thermodynamics, German Aerospace Center, Pfaffenwaldring 38-40, Stuttgart, 70569, Germany*

[2]*Hydrogenics Corporation, 220 Admiral Boulevard, Mississauga, ON L5T 2N6, Canada*

[3]*Institute of Materials Research, German Aerospace Center, Linder Hoehe, 51147, Cologne, Germany*

[4]*University of Applied Sciences Esslingen, Dept. of Basic Science, Kanalstrasse 33, 73728, Esslingen, Germany*

[5]*Institute of Energy Storage, University of Stuttgart, Stuttgart, 70550, Germany*

*E-Mail: aldo.gago@dlr.de (A. S. Gago)

SEM before electrochemical measurements

The SEM pictures of figure S1 show the Nb/Ti/ss, Nb/Ti and Nb/ss samples. The pictures focus on the well-covered surface by the niobium PVD coating before the accelerated stress test (AST). The coating is approximately 1 µm thick and was produced on the vacuum plasma sprayed Ti coating as well as on a pure Ti sheet and stainless steel 316L respectively.

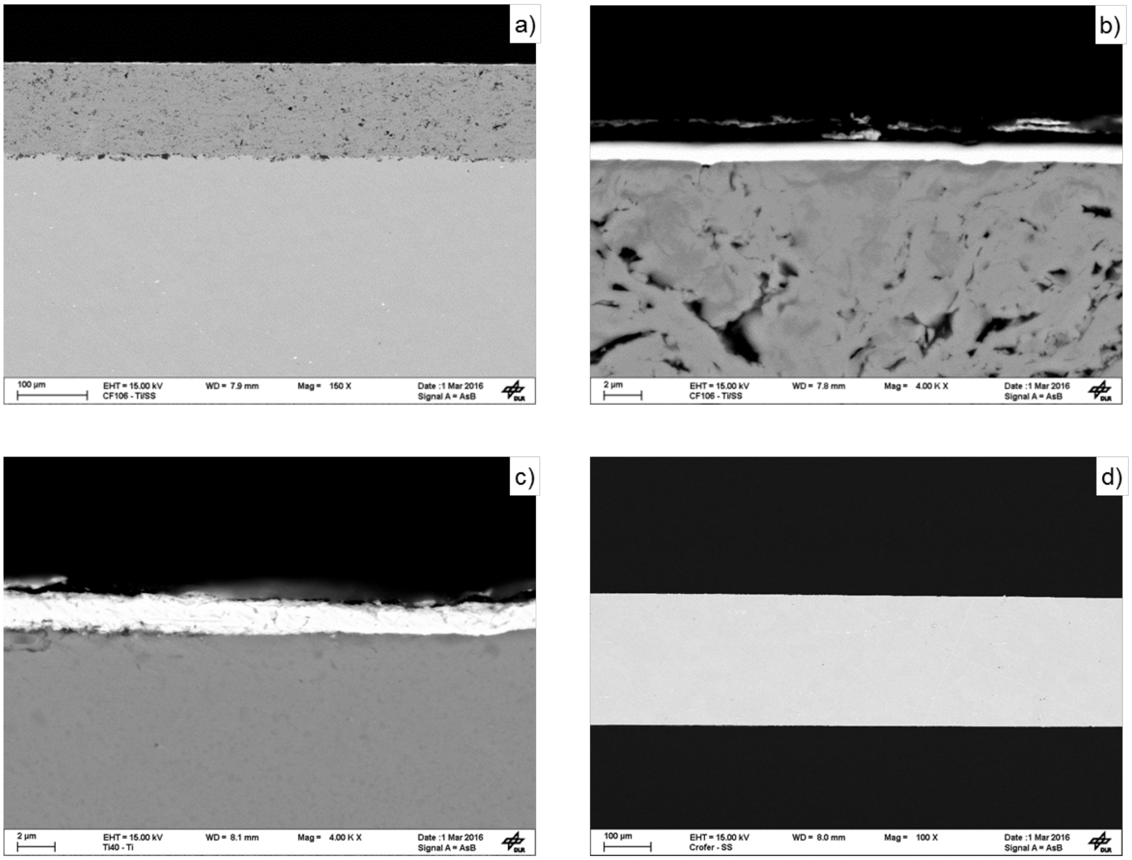

Figure S1: a) Pristine overview cross-section SEM picture of the Nb/Ti/ss sample, b) zoomed in to the Nb/Ti boundary layer of Nb/Ti/ss. c) zoomed in to the Nb/Ti boundary layer of Nb/Ti. d) Overview cross section SEM picture of Nb/ss sample.

Metal/Oxide ratio determined by XPS

Figure S2 depicts the analysis of the depth profiles of the niobium coating made by XPS during Ar- sputtering. The depth profile shows a significant growing of the oxide layer. Also the overall metal- oxide ratio decreases in the whole niobium layer.

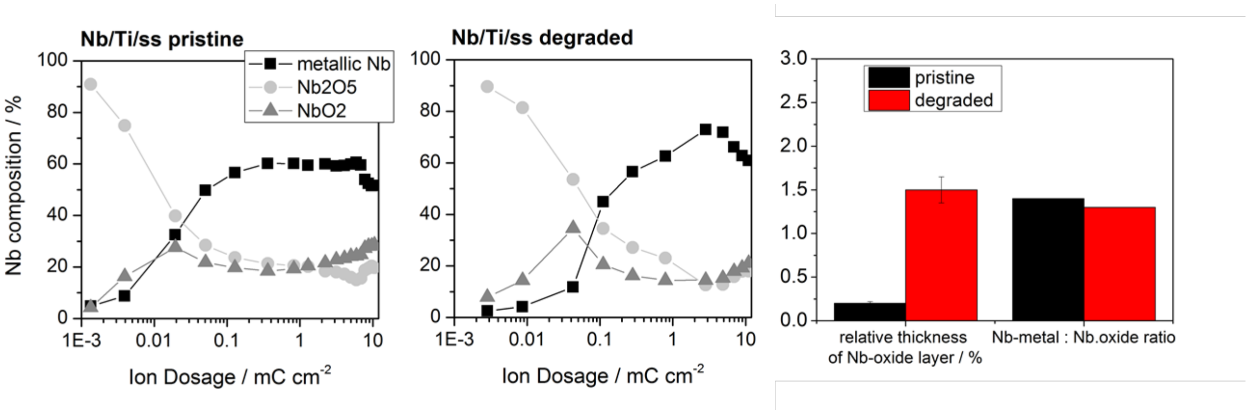

Figure S2: Left: pristine depth profile analysis of niobium on the Nb/Ti/ss sample; middle: depth profile analysis of niobium on the Nb/Ti/ss sample after electrochemical measurements. Right: Ratio of relative thickness of Nb-oxide layer in percent and Nb-metal vs. Nb-oxide of the pristine and the sample of Nb/Ti/ss after electrochemical tests.

SEM after electrochemical measurements

The SEM pictures of Figure S3 show the Nb/Ti/ss, Nb/Ti and Nb/ss samples, after the AST in the 0.5 M H_2SO_4 at 65°C. The detached layer in Figure S3b is not necessary due to electrochemical AST but most probably from the preparation method. Obvious is the leached out region below the PVD niobium coating in Picture S3d. It shows clearly, that the PVD coating alone is not able to protect the stainless steel at potentials around 2 V for more than 6 h while at the Ti-coated stainless steel sample (Figure S3a and b) no changes could be observed after AST.

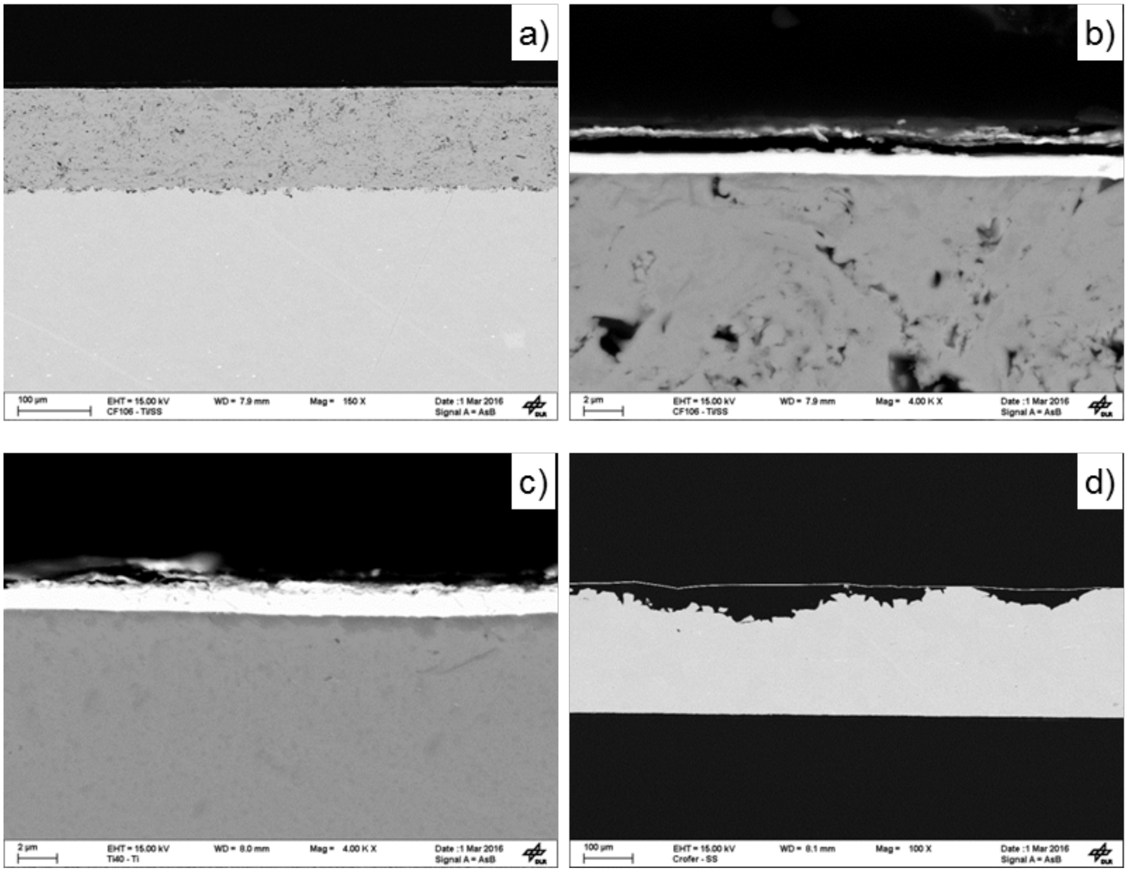

Figure S3: a) Overview cross section SEM picture of Nb/Ti/ss sample, b) zoomed in to the Nb/Ti boundary layer of Nb/Ti/ss. c) zoomed in to the Nb/Ti boundary layer of Nb/Ti. d) Overview cross section SEM picture of Nb/ss sample. All samples after electrochemical tests.

AFM analysis

On the Nb/Ti/ss samples, no evidence of corrosion or damage of the Nb coating was detected. No change in conductive area before (Figure S4.2 b) and after ecm was observed (Figure S4.2 d) at an applied voltage of 3 V. The surface of the Ti protected samples stayed without change in contrast to the samples with an Nb layer coated on ss.

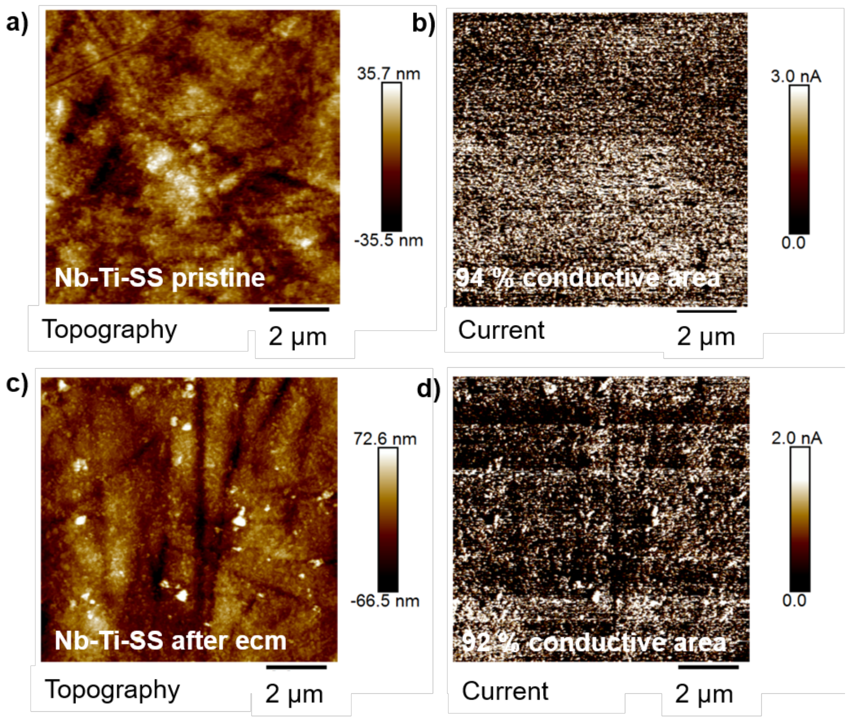

Figure S4.2: AFM images of pristine Nb/Ti/ss, a) topography, and b) corresponding current, and after ecm c) topography, and d) corresponding current.

The AFM topography measurements of the Nb coating on SS before electrochemical measurement (ecm) is shown in 4.3 a) and c), and as a close-up view in d). A continuous layer of small Nb crystals with approximately 50 nm width, visible in Figure 2d), was measured. At an applied voltage U=3 V between sample and AFM tip, the Nb/ss surface was almost completely conductive (Figure S4.2b). The remaining non-conductive area of 7 % remained was most probably caused by impurities on the sample surface. After ecm, a larger scale pitting corrosion area was observed by optical microscopy. With AFM, also smaller pinholes as shown in Figure S4.2e were measured. Pitting corrosion on stainless steel (ss) was also visible at small defects of the Nb layer. Around damage the pit, a non-conductive area, most-probably non-conductive corrosion products, were found (Figure S4.2f). In contrast, after the ecm at many spots the conductivity was higher, even at a lower applied voltage. A possible explanation could be breakage of the Nb layer during ecm due to corrosion creep.

On samples containing additional Ti, topography and conductivity measurements before ecm are shown in Figure S4.2g and S4.2h. After ecm, a decrease in conductive area was determined at an applied voltage of U=3 V (Figure S4.2i and j). From the topographic images, no evidence of pitting corrosion was found.

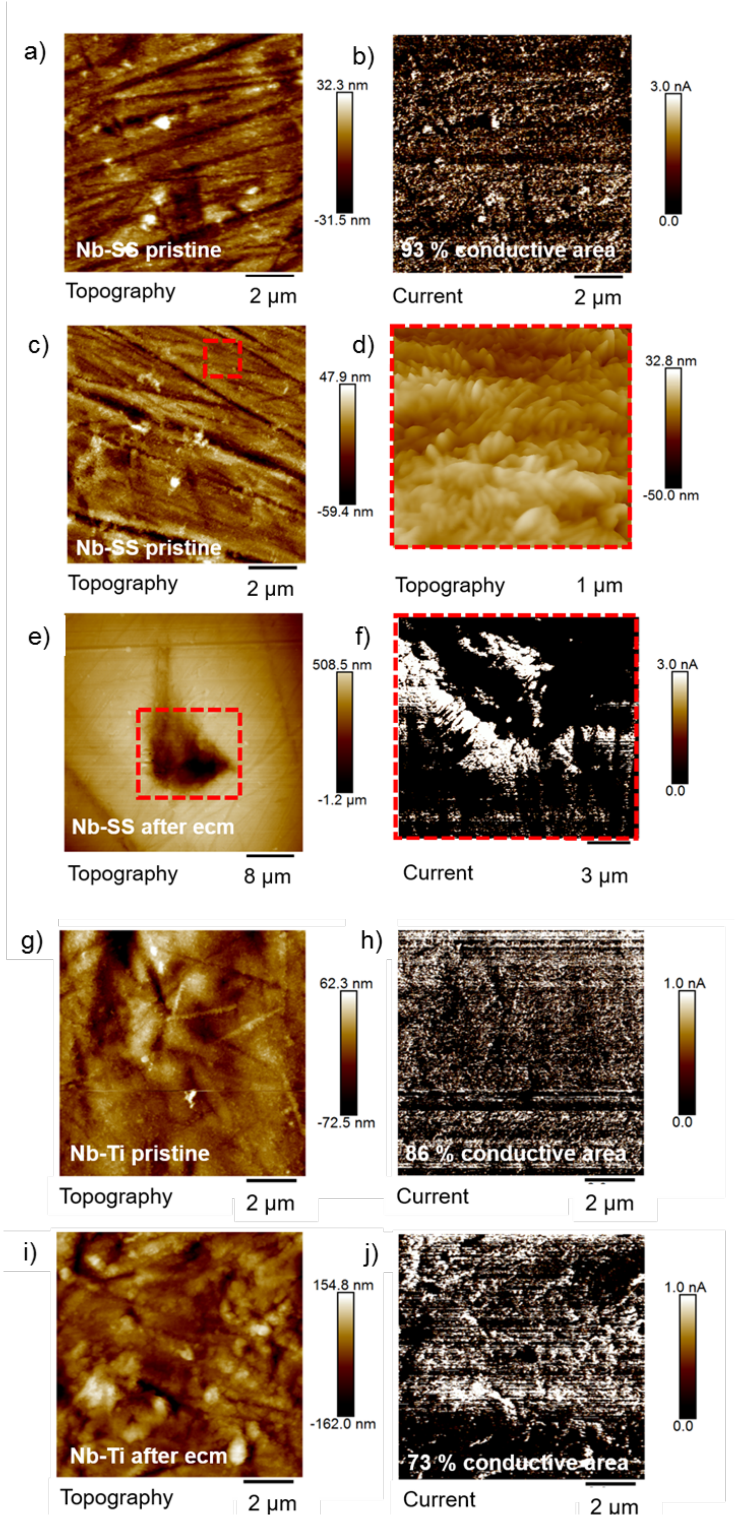

Figure S4.3: AFM images of pristine Nb/ss, a) topography, b) corresponding current measurement, c) topography of pristine Nb/ss, d) zoom of c) as 3D-view, e) AFM topography image of pitting corrosion on the Nb/ss sample after ecm, and f) corresponding current

measurement. AFM images of pristine Nb/Ti, g) topography, and h) corresponding current image. AFM images of Nb-Ti after ecm i) topography, and j) current.

PEM electrolyzer cell performance

Figure S5a shows the polarization curves at the initial state, after 500 and 1000 h constant operation at 1 A cm^{-2} of cell 1 and cell 2 respectively. While both cells have Nb/Ti as an anode site coating on stainless steel based BPP, cell 1 has Pt/Ti and cell 2 Nb/Ti as a cathode site coating. The performance over time is getting better due to activation processes of the membrane, and electrode aging of the anode. This was reported previously and is stable after 500 h.[1] Figure S5b show the performance of the two coated cells compared to a commercial bulk Ti produces state of the art cell, which has an ICR reducing coating as well. Even if a direct comparison is not possible due to the use of different types of MEAs, since the recorded UI curves are made in the same short stack, it can be used as a preliminary comparison to what is on the market. Cell 2 is due to the high ohmic resistance, which is caused by the cathode site coating not comparable, but cell 1 is indeed.

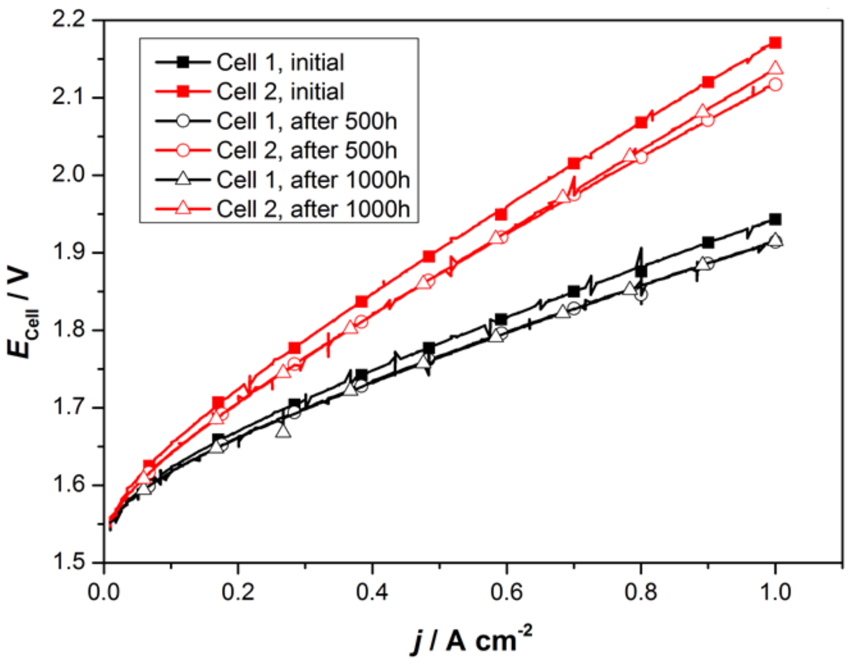

Figure S5a: a) current potential curve of cell 1 and cell 2 before and after the 500 long term test and after 1000 h, from 0.01A cm^{-2} to 1 A cm^{-2} at ca. 29°C and a scanning rate of 4mA cm^{-2} s^{-1}. b) initial potential curve of cell 1 and cell 2 and the Ti-based reference cell with surface coating, from 0.01A cm^{-2} to 1 A cm^{-2} at ca. 29°C and a scanning rate of 4 mA cm^{-2} s^{-1}.

Electrochemical analysis of EIS

The analysis of the EIS measurements was carried out using an equivalent circuit presented in Figure S5b. It consists of an ohmic resistor representing the ohmic behaving components such as ionomer and electric interconnectors followed by two (R/CPE) elements due to the two time constants which can be observed for the impedance spectra. CPE is a non-ideal capacitor

represented by $j\omega^n$ while n is between 0 and 1 and 1 is used for an ideal capacitor. The low frequency (LF) arc represents the rate determining charge transfer of the oxygen evolution reaction. The high frequency arc is not yet well interpreted. Some groups report the HF arc representing the fast reaction of the hydrogen evolution reaction (HER).[2,3] Others call it charge transfer resistance of ionic and ohmic components in the active part of the electrode combined with double layer effects or the first electron transfer of the two electron step reaction of the OER.[4–7]

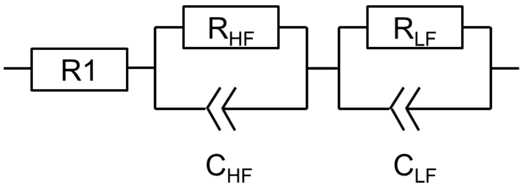

Figure S5b: equivalent circuit for analyzing the recorded electrochemical impedance spectra.

By using only the analyzed parameter of the equivalent circuit presented in Figure S5b, the polarization curve of each cell can be simulated and are presented in figure S5c. The temperature and pressure profiles of the measured polarization curves were used as input parameters for the simulation. For cell 1 the data fit fairly well, which proves its consistency with the EIS measurements and the polarization curves, even though these were recorded at different temperatures. Cell 2 fits less than Cell 1. However Cell 2 is in contact with the end plate, which may cause temperature differences compared to the temperature values recorded at the anode side outlet of the opposite end plate. Cooling effects due to the end plate can cause the higher potentials of cell 8 and the small deviation of the ohmic resistance measured by EIS and the ohmic resistance resulting in the slope of the polarization curve of cell 2.

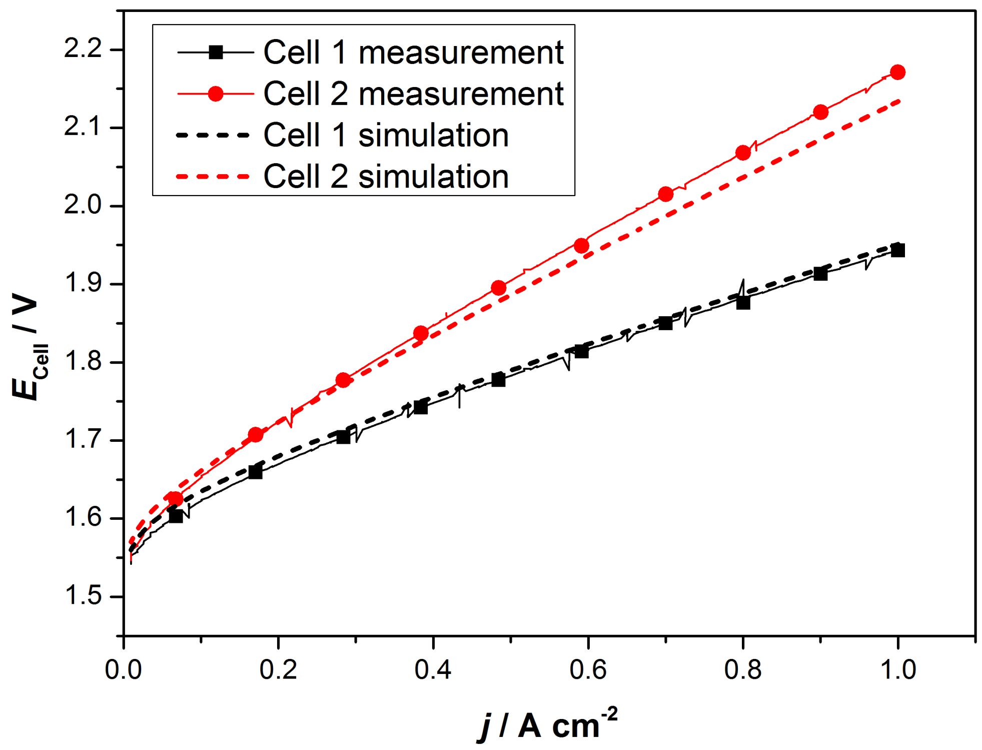

Figure S5c: Measurements and simulation of cell 1 and cell 2 as well as the simulation of both cells based on the analysis of EIS and using the pressure and temperature profiles.

SEM and ICR after long-term test in PEM electrolyzer

The SEM pictures of Figure S6 show a) the Nb/Ti-coated stainless steel bipolar plate after 1000h of operation, b) zooms to the anode site and c) to the cathode site. The thin white coating which seems to hang in the middle of the flow field is the niobium PVD coating which sticks on the artifact which is left from the post treatment of the VPS coating. Figure S6b shows the good contact to the titanium layer while figure S6c depicts the separation of the two coatings which we think is coming by hydrogen embrittlement in combination of oxidation of the Niobium.

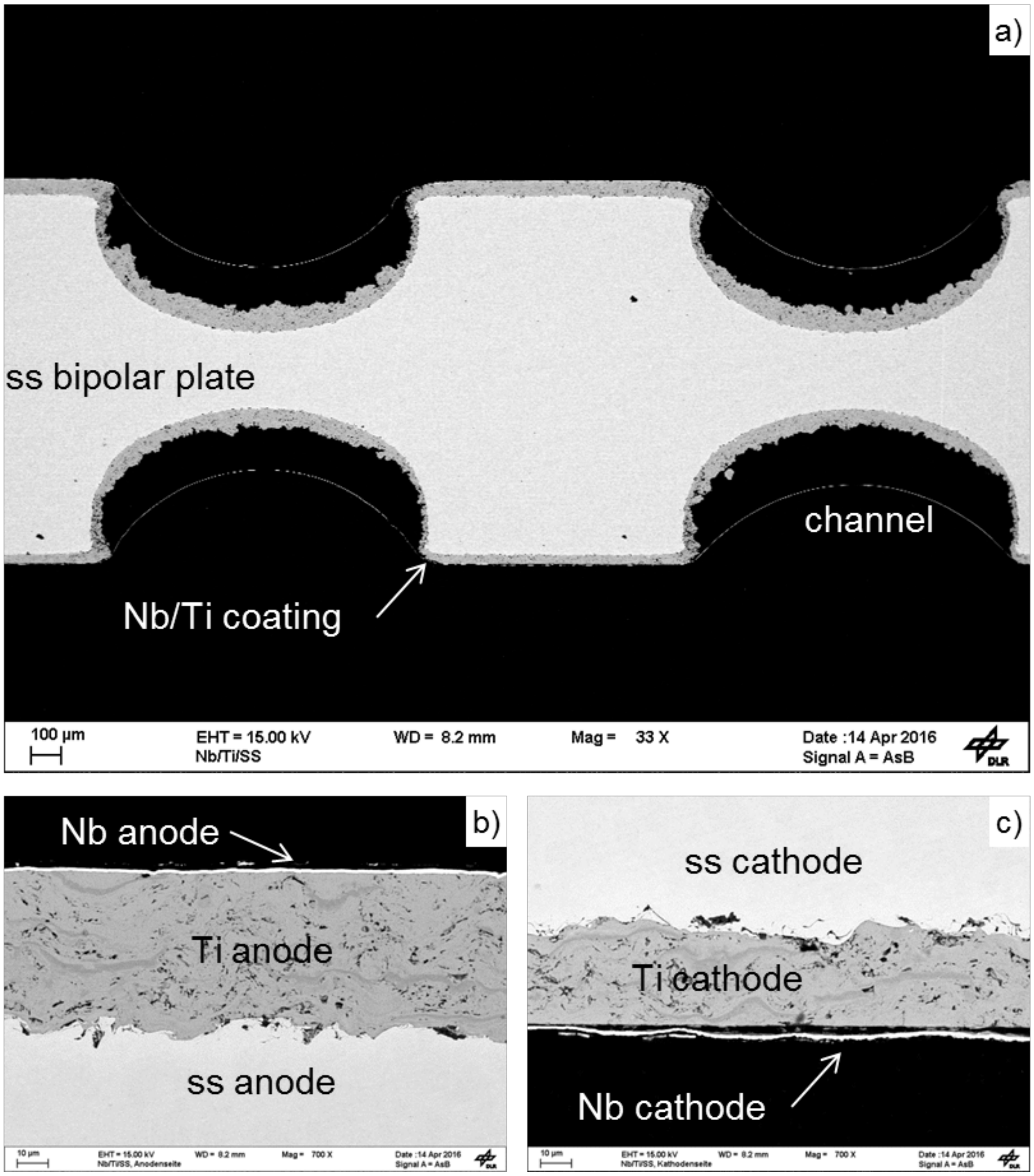

Figure S6: a) Overview cross section SEM picture of Nb/Ti/ss bipolar plate after 1000h at constant current of 1 A cm^{-2}, 38°C and 95 psi. b) zoomed in to the anode of this BPP. c) zoomed in to the cathode.

ICR measurements

The schemes in Figure S7.2 and S7.3 illustrate the experimental setup for the ICR measurements. Their purpose is to indicate the differences between measuring the flat samples used for corrosion measurements and BPPs with flow field. The flow field design shown in the scheme does not correspond to the one in the 92E stack.

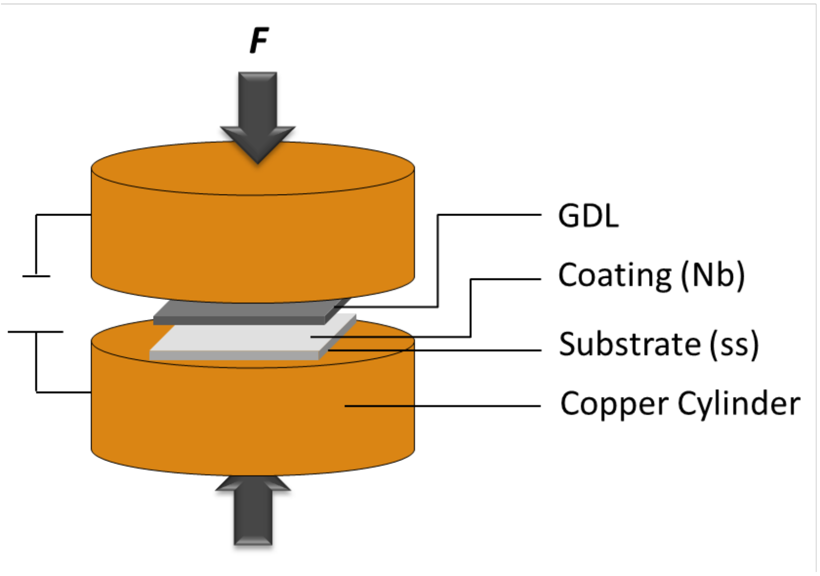

Figure S7.1: Scheme of the ICR measurement setup of coating samples

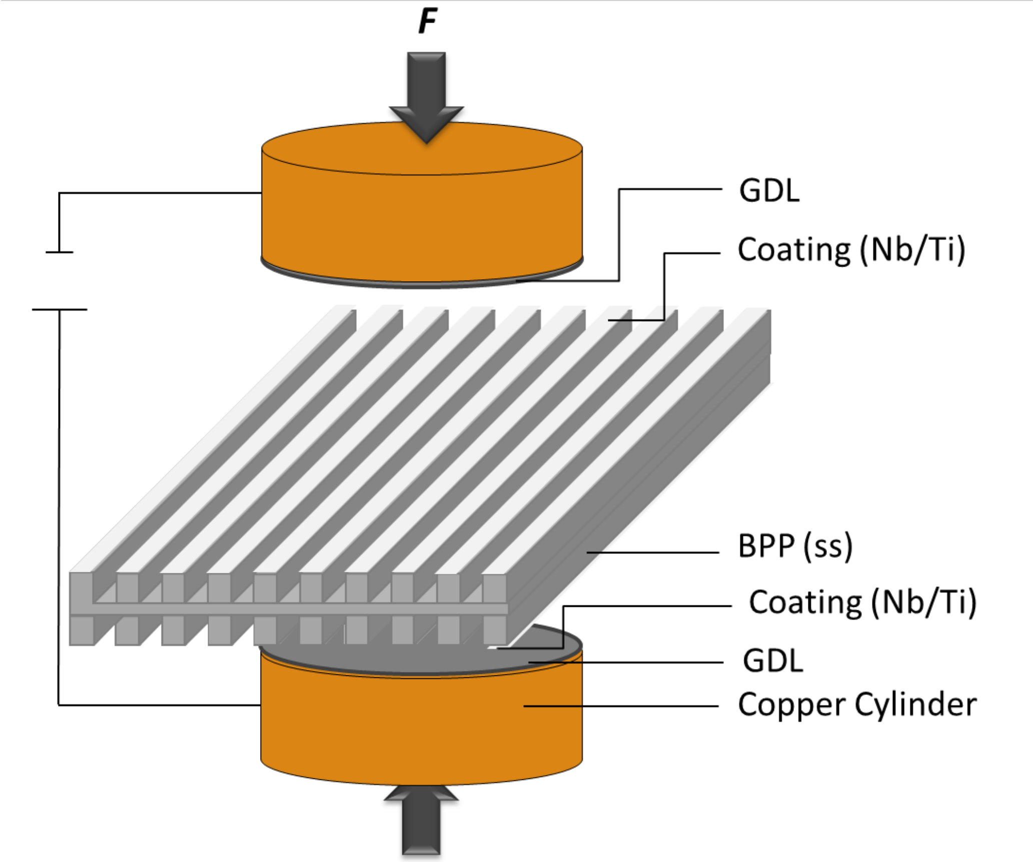

Fig S7.2: Scheme of the ICR measurement setup of coated bipolar plates

The Measurements were carried out by measuring the potential answer at 5A and successively increased pressure and normalized by surface. The surface for the bipolar plate ICR measurements is the surface of the GDL divided by 2 in order to correct the flow field impact.

$$R = A\frac{U}{I}$$

$$R_{surface} = R_{measurement} - R_{offset}$$

Post mortem ICR

Figure S7 shows the ICR measurements of the bipolar plates after 1000 h of operation. To depict the influence of the surface oxidation we sanded the anode site and the cathode site. The only significant difference can be observed after sanding the cathode site of plate 2, which is in fact the cathode site of cell 2. The cathode site of plate 2 was not in use for this assembly while in contact to the end plate.

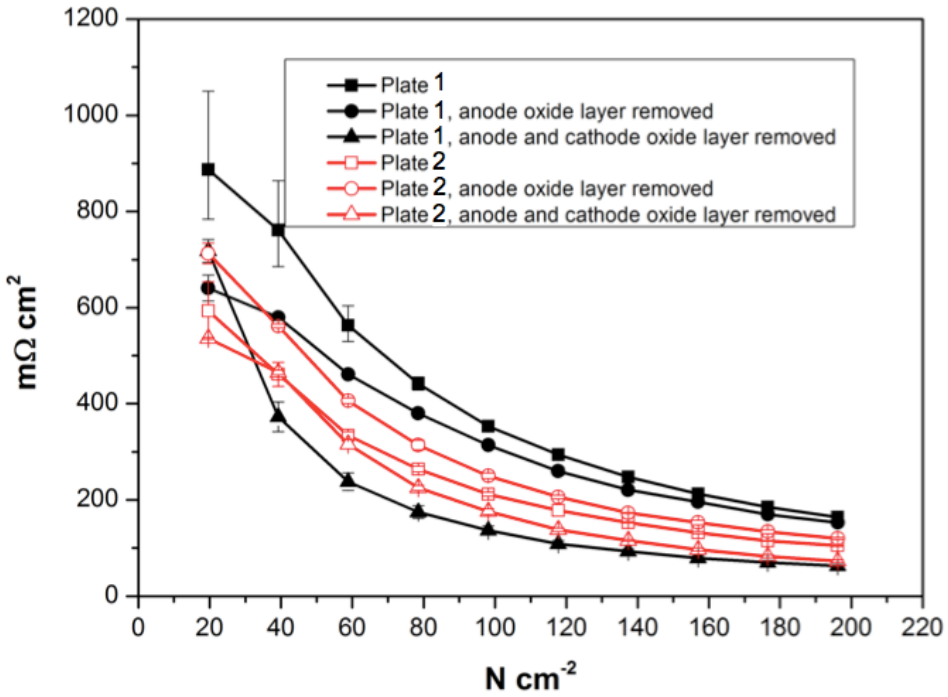

Figure S7: Interfacial contact resistance vs. compact force in N cm^{-2} of Nb/Ti/ss bipolar plate after 1000h long term test at constant current of 1 A cm^{-2}, 38°C and 95 psi. Stepwise sanded, first anode than the cathode site as well.

Commissioning of the stack

Figure S8 presents the very first measurement points of the used short stack. It can be observed, that during the first seconds the potential of cell 2 increases rapidly. This indicates, that degradation mechanism occur during the first minutes, which could give a explanation of the performance differences of cell 2 and cell 1 after the activation protocol but before the 1000 h

long term test. Additionally, this data are not necessary the very first in general since stacks gets usually first commissioned by the manufacturer.

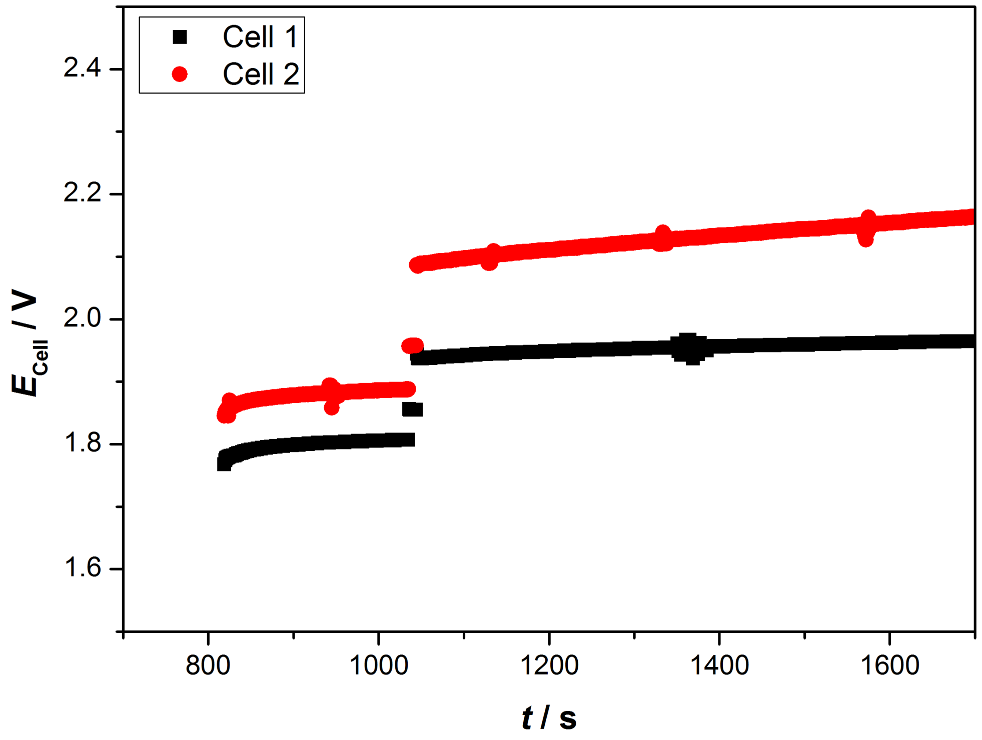

Fig. S8. Cell voltages *vs.* time of the first measurement points of the 92E short stack at two different current densities at 25 °C.

References

1. Lettenmeier, P. *et al.* Durable Membrane Electrode Assemblies for Proton Exchange Membrane Electrolyzer Systems Operating at High Current Densities. *Electrochim. Acta* **210,** 502–511 (2016).
2. Travassos, M. a., Lopes, V. V., Silva, R. a., Novais, a. Q. & Rangel, C. M. Assessing cell polarity reversal degradation phenomena in PEM fuel cells by electrochemical impedance spectroscopy. *Int. J. Hydrogen Energy* **38,** 7684–7696 (2013).
3. Malevich, D., Halliop, E., Peppley, B. a., Pharoah, J. G. & Karan, K. Investigation of Charge-Transfer and Mass-Transport Resistances in PEMFCs with Microporous Layer Using Electrochemical Impedance Spectroscopy. *J. Electrochem. Soc.* **156,** B216 (2009).
4. Rozain, C. & Millet, P. Electrochemical characterization of Polymer Electrolyte Membrane Water Electrolysis Cells. *Electrochim. Acta* **131,** 160–167 (2014).
5. Antoine, O., Bultel, Y. & Durand, R. Oxygen reduction reaction kinetics and mechanism on platinum nanoparticles inside Nafion®. *J. Electroanal. Chem.* **499,** 85–94 (2001).
6. Mueller, J. T. & Urban, P. M. Characterization of direct methanol fuel cells by ac impedance spectroscopy. *J. Power Sources* **75,** 139–143 (1998).
7. Eikerling, M. & Kornyshev, a. a. Electrochemical impedance of the cathode catalyst layer

in polymer electrolyte fuel cells. *J. Electroanal. Chem.* **475,** 107–123 (1999).
